# Comparative oncology – the North American experience

**DOI:** 10.1186/1753-6561-7-S2-K5

**Published:** 2013-04-04

**Authors:** David M Vail

**Affiliations:** 1 School of Veterinary Medicine and the Carbone Comprehensive Cancer Center, University of Wisconsin-Madison, Madison, WI 53706, USA

## 

Comparative oncology can be used to describe a discipline that integrates the study of naturally occurring cancers in animals into studies of human cancer biology and therapy. The term is most often used when referring to the study of cancers seen in companion (pet) animals. Cancers in companion species are well suited to uniquely inform investigations of cancer biology and drug development. Clinical trials in veterinary oncology have gained interest and focus over the last decade, with both veterinary and comparative oncology drug development leading the effort. There have also been a growing number of consortia and cooperative groups that have functioned successfully by uniting multiinstitutional efforts, advocating for veterinary clinical trials, and emphasizing the synergy between basic science and clinical progress. With support from clients who are motivated to seek advanced care for their pets and to enroll them in investigational trials that offer new therapies, clinical research in veterinary oncology is growing in scope and importance.

Oncology clinical trials attempt to answer questions and find better ways to prevent, diagnose, or treat cancer. Their model is different from trials involving infectious or even chronic diseases because the risks involved have greater morbidity and mortality. Traditional drug development follows a strict, step-wise paradigm that begins with a phase I dose-finding trial, followed by a phase II efficacy/activity trial, and concludes with a phase III "pivotal" trial that pits a novel agent against or with the current standard of care (Table 1). Although veterinary oncology trials sometimes combine these concepts, their individual descriptions serve as the framework for new drug development. Clinical designs, pertinent endpoints and analyses, the process for drug approval, and clinical trial ethics will be explored in the following sections.

**Table 1:** Goals of Phase I–III Clinical Trials

CHARACTERISTIC	PHASE OF CLINICAL TRIAL		
	
	PHASE I (DOSE FINDING)	PHASE II (ACTIVITY/EFFICACY)	PHASE III (PIVOTAL)
Primary goals	• Determine MTD • Define DLT • Characterize type and severity of adverse events	• Determine activity/efficacy in defined populations • Inform the decision to move to a Phase III trial	• Compare a new drug or combination to therapy currently regarded as standard of care

Secondary goals	• PK/PD issues • Scheduling issues • Target modulation effects • Preliminary efficacy data • Investigate surrogate biomarkers of response	• Estimate therapeutic index • Expand adverse event data • Evaluate additional dosing groups • Expand target modulation and biomarker data • Quality of life measures	• Quality of life comparisons • Comparative costs

MTD, Maximum tolerated dose; DLT, dose-limiting toxicity; PK/PD, pharmacokinetic/pharmacodynamic.

## Consortia

One of the most exciting achievements in veterinary oncology over the last decade has been the development of successful and collaborative consortia groups that are purposed to perform multicenter clinical trials and prospective tumor biospecimen repository collections. Consortia infrastructures allow larger scale clinical trials and provide the voice for collective advocacy in veterinary and comparative oncology. Their success is an example of the growing importance of the study of tumor and clinical biology. Some of these efforts are profiled here.

## Comparative Oncology Trials Consortium

The Comparative Oncology Trials Consortium (COTC) is an active network of 20 centers (https://ccrod.cancer.gov/confluence/display/CCRCOPWeb/Comparative+Oncology+Trials+Consortium), centrally managed by the National Institutes of Health-National Cancer Institute's Comparative Oncology Program (NCI-COP), that functions to design and execute clinical trials in dogs with cancer to assess novel therapies. The goal of this effort is to answer biologic questions geared to inform the development path of these agents for use in human cancer patients. As of 2012, the COTC has completed or is currently conducting 18 clinical trials and has been successful in promoting the utility of comparative oncology modeling within the drug development community.

## Animal Clinical Investigation

Animal Clinical Investigation (ACI; http://www.animalci.com) is a privately organized and run specialty network of veterinary hospitals that designs, conducts, and reports clinical studies for the animal health industry. ACI trials emphasize oncology drug development but have recently expanded to include other medical conditions, including inflammatory and metabolic disease, cardiovascular disease, and arthritis. ACI provides multi-site, pivotal, or nonpivotal studies and commercialization support to help define effective novel veterinary therapeutics. It is the first example of a commercial clinical research organization (CRO) in veterinary medicine. Over 30 clinical trials have been conducted through this network over the last 10 years.

## Canine Comparative Oncology and Genomics Consortium

The Canine Comparative Oncology and Genomics Consortium (CCOGC; http://www.ccogc.net) is an informal collaboration of veterinary and medical oncologists, pathologists, surgeons, geneticists, and molecular and cellular biologists with a common interest in the comparative study of canine and human genomics and cancer. The goals of the CCOGC are to facilitate partnerships and collaborations focused on the problem of cancer in dogs. Early priorities included advocacy for the field of comparative oncology, the development of a mechanism to share reagents and resources in the community, and the development of a biospecimen repository. The repository houses tumor tissue, normal tissues, serum, plasma, peripheral blood mononuclear cell preparations, genomic DNA, RNA, and urine samples. As of 2011 the CCOGC has collected 1600 of 3000 anticipated samples. The repository will serve as a seminal resource for the research community to acquire tissues for target biology studies, as well as its mechanism for prospective collections of unique samples. Future efforts will focus on dissemination of these tissues.

## The traditional drug development flow

Traditionally, *first-in-species trials* start with a *Phase I* dose-finding trial, followed by a *Phase II* efficacy/activity trial and concluding with a *Phase III* “comparative” trial that pits the novel agent against or with the current standard of care (Table 1).

## Conclusions

Clinical trials are an important research discipline to improve care and outcome for cancer patients in both human and veterinary oncology. Steps should be made to ensure study aims are achievable within a crafted study design and protocol. Rules governing design are prospective and involve questions of dose and schedule selection, toxicity, activity, and comparison to known effective therapies. Statistical expertise is also necessary to ensure appropriate clinical trial design. Regulatory oversight of veterinary oncology trials is increasing, and new approval of veterinary oncology agents will emphasize these processes over the next decade. Comparative oncology trials also are key to the inclusion of pet animals in the evaluation of novel anticancer therapeutics, imaging strategies, and medical devices. Consortia groups will continue to advocate and advance the use and utility of veterinary oncology clinical trials. The field of veterinary oncology will continue to grow through the proper use and design of both traditional and novel clinical trials.

